# Women with gynecologic cancers need more psychological attention during the COVID-19 pandemic

**DOI:** 10.17179/excli2021-3364

**Published:** 2021-02-01

**Authors:** Leila Allahqoli, Mojgan Karimi-Zarchi, Hamid Salehiniya, Ibrahim Alkatout

**Affiliations:** 1 Iran University of Medical Sciences (IUMS), Tehran, Iran; 2 Department of Gynecology Oncology, Firoozgar Hospital, Iran University of Medical Sciences, Tehran, Iran; 3 Social Determinants of Health Research Center, Birjand University of Medical Sciences, Birjand, Iran; 4 University Hospitals Schleswig-Holstein, Campus Kiel, Kiel School of Gynaecological Endoscopy. Arnold-Heller-Str. 3, Haus 24, 24105 Kiel, Germany

## ⁯

***Dear Editor,***

The outbreak of the coronavirus disease (COVID-19) in December 2019 (WHO, 2020[11]) marked the beginning of a pandemic that affected health care services across the globe (Tsai et al., 2020[9]). The disease was interpreted as a major threat to public health in late 2019. Fear of being infected by COVID-19, global lockdowns, disruptions in transportation (Shoukat et al., 2020[8]), and the allocation of existing resources to the care of COVID-19 patients disrupted the delivery of medical services to cancer patients (Gong et al., 2020[5]). The interruption of services in oncology has led to higher mortality rates, increased the disease burden and the economic burden, and also affected the patients' psychosocial functioning and emotional well-being (Rothan and Byrareddy, 2020[7]). Cancer patients experience immunosuppression due to their disease as well as its treatment. These patients are highly vulnerable to psychological distress during the COVID-19 pandemic (CDC, 2020[4]). We believe that, during the pandemic, a number of factors have rendered women with gynecologic cancers more susceptible to psychological distress than patients with other types of cancer.

Female cancer patients are known to suffer greater psychological distress than their male counterparts (Parás-Bravo et al., 2020[6]). Women with gynecologic cancers are vulnerable to psychological distress because of fertility concerns and the fear of losing their partner. The patients are also prone to neurocognitive dysfunction, which leads to adverse mental health outcomes (Andryszak et al., 2017[2]). Such distress may be followed by high levels of depression, anxiety, and post-traumatic stress disorders (PTSD) in conjunction with poor quality of life (QoL) (Cassedy et al., 2018[3]; Warren et al., 2018[10]). 

In view of the above concerns, women with gynecologic cancers require more attention in the COVID-19 crisis. The following measures are recommended to reduce the deleterious effects of the pandemic:

- Gynecologic oncologists should include a psychiatrist and a psychologist in their team in order to aid patients in coping more effectively with their disease and mental health problems.
- Distress levels should be measured in a structured manner. We advise the use of effective and standardized screening tools for early detection and management of distress in order to reduce the disease burden.
- Social support and supportive care by the family or health professionals must exceed the levels provided under normal circumstances in order to create a buffer against psychological distress. Family members play an important role in relieving psychological difficulties and should devote more time to the care of the patients. Family members should also be skilled in reporting symptoms to health care professionals. The latter will then be able to make appropriate and timely decisions.
- Partners and families of cancer patients should be trained in, or acquire, basic skills (mental health rehabilitation services) to provide adequate support for patients, relieve their fear and anxiety, and address their mental health issues.
- Effective communication models should be developed to facilitate communication between healthcare professionals, patients, and their partners.
- Patients should be better informed and empowered. In addition to being involved in the treatment, their preferences, values and needs should be taken into account.
- Appropriate interventions such as education, case management, the chronic care model (CCM), multidisciplinary teams (MDT), complex interventions, exercise, and art therapy should be designed to address the patients' concerns and improve their quality of life.

As women with gynecologic cancer are highly susceptible to experiencing mental health problems, their treatment should be addressed by a multidisciplinary team. A successful physician-patient relationship forms the foundation of effective treatment (Alkatout, 2018[1]). 

## Conflict of interest

The authors declare no conflict of interest.
